# Feasibility of oropharyngeal and respiratory muscle training in individuals with OSA and spinal cord injury or disease: A pilot study

**DOI:** 10.14814/phy2.15930

**Published:** 2024-02-07

**Authors:** Abdulghani Sankari, Abed Alkader Najjar, Scott A. Maresh, Joel L. Prowting, Constance H. Fung, Arthur Knack, Hossein Yarandi, M. Safwan Badr

**Affiliations:** ^1^ Department of Medicine, John D. Dingell VA Medical Center Detroit Michigan USA; ^2^ Department of Medicine Michigan State University East Lansing Michigan USA; ^3^ Department of Medicine Wayne State University Detroit Michigan USA; ^4^ Department of Medical Education, Ascension Providence Medical Center Southfield Michigan USA; ^5^ School of Kinesiology and Health Science York University Toronto Ontario Canada; ^6^ Department of Medicine UCLA David Geffen School of Medicine California Los Angeles USA; ^7^ Geriatric Research, Education and Clinical Center, VA Greater Los Angeles Healthcare System California Los Angeles USA

**Keywords:** obstructive sleep apnea, pulmonary function, spinal cord injury; respiratory muscle training

## Abstract

**Objectives:**

To examine the feasibility of individuals with spinal cord injury or disease (SCI/D) to perform combined oropharyngeal and respiratory muscle training (RMT) and determine its impact on their respiratory function.

**Methods:**

A prospective study at a single Veterans Affairs (VA) Medical Center. Inclusion criteria included: 1) Veterans with chronic SCI/D (>6 months postinjury and American Spinal Injury Association (ASIA) classification A–D) and 2) evidence of OSA by apnea‐hypopnea index (AHI ≥5 events/h). Eligible participants were randomly assigned to either an experimental (exercise) group that involved performing daily inspiratory, expiratory (using POWERbreathe and Expiratory Muscle Strength Trainer 150 devices, respectively), and tongue strengthening exercises or a control (sham) group that involved using a sham device, for a 3‐month period. Spirometry, maximal expiratory pressure (MEP), maximal inspiratory pressure (MIP), polysomnography, and sleep questionnaires were assessed at baseline and at 3 months.

**Results:**

Twenty‐four individuals were randomized (12 participants in each arm). A total of eight (67%) participants completed the exercise arm, and ten (83%) participants completed the sham arm. MIP was significantly increased (*p* < 0.05) in the exercise group compared with the baseline.

**Conclusions:**

Combined oropharyngeal and RMT are feasible for individuals with SCI/D. Future studies are needed to determine the clinical efficacy of these respiratory muscle exercises.

## 1 INTRODUCTION

Veterans with spinal cord injuries and disorders (SCI/D) commonly experience impairments in physiological function, activities of daily living, and long‐term health (Sankari et al., 2019). Since respiratory impairment following SCI/D is typically more profound in the supine position (Sankari et al., 2014), positive airway pressure (PAP) therapy is often recommended (Daoud et al., 2020). However, adherence to PAP therapy remains a challenge despite education, follow‐up, and support (Sawyer et al., 2011). Because patients with SCI may be less adherent to PAP therapy than nondisabled individuals (Sankari et al., 2015), alternative treatment approaches are needed. Oropharyngeal and respiratory muscle training (RMT) has promising potential for treating breathing disorders (Guimarães et al., 2009) in patients with SCI/D. RMT typically includes specific repetitive breathing exercises with hand‐held respiratory trainer devices to provide pressure threshold or flow‐dependent resistance against inspiratory, expiratory, or both to improve both muscular strength and endurance. The goal of a typical training session is to complete a certain number of exercises for a particular length of time.

The principles underpinning effective training of the respiratory muscles are similar to other skeletal muscles, requiring progressive increases of load over time in order to stimulate adaptation (MÀC et al., 2018). Studies have suggested that training the respiratory accessory muscles in persons with tetraplegia improves respiratory muscle strength (as manifested by significant improvement in maximal inspiratory MIP and expiratory pressures MEP; respectively) and respiratory symptoms (Berlowitz & Tamplin, 2013; Roth et al., 2010; Rutchik et al., 1998). Previous studies have shown that oropharyngeal and RMT exercises reduce the severity of obstructive sleep apnea (OSA) in able‐bodied individuals (Guimarães et al., 2009). Others have demonstrated that RMT improves inspiratory and expiratory strength in patients with cardiac disease (Mancini et al., 1995), respiratory disorders (Berry et al., 2012), and SCI/D (Berlowitz & Tamplin, 2013), suggesting a potential therapeutic role in patients with SCI/D. However, the feasibility of RMT combined with oropharyngeal training exercise and its effects on sleep are unknown. Therefore, the objectives of this study are to (1) determine the feasibility of combined oropharyngeal and RMT exercise in Veterans with OSA and SCI/D and (2) determine its impact on their respiratory function and acceptability of the exercise.

## 2 METHODS

### 2.1 Study design and ethics

This study utilized a single‐center randomized, controlled, and single‐blinded design. The Human Investigation Committees of Wayne State University and Detroit Veterans Affairs (VA) Medical Center approved the experimental protocols (#100712M1F(V)), and the study was preregistered on Clinicaltrials.gov NCT03664765. Written informed consent was obtained from all participating subjects.

### 2.2 Recruitment and screening

The study targeted Veterans who receive SCI/D care at a single local VA medical center and its catchment area to assess for eligibility to be screened for inclusion in the study. The duration of recruitment for this study was between October 1, 2018 and May 31, 2021. Inclusion criteria were (1) Veterans with chronic SCI/D (>6 months postinjury and American Spinal Injury Association (ASIA) classification A‐D) (i.e., excluding those with no evidence of a neurologic deficit based on ASIA classification) and (2) apnea‐hypopnea index (AHI) ≥5 events/h. Exclusion criteria were (1) receiving continuous mechanical ventilation (except PAP therapy which is considered a usual treatment for sleep‐disordered breathing (SDB), (2) severe congestive heart failure with ejection fraction <35%, (3) recent health event that may affect sleep (e.g., stroke, acute myocardial infarction, recent surgery, or hospitalization), (4) alcohol or substance abuse (<90 days sobriety), (5) self‐described as too ill to engage in study procedures, (6) unable to provide self‐consent for participation (e.g., due to dementia), and 7) central sleep apnea (CSA) defined as central apnea/hypopnea >50% of the AHI.

#### 2.2.1 Baseline assessment

Each participant underwent a full in‐laboratory polysomnography (PSG). PSGs were performed according to AASM standards and respiratory events were scored according to the American Academy of Sleep Medicine (AASM) recommended scoring criterion (Berry et al., 2012). A bedside baseline spirometry and respiratory muscle forces (maximal inspiratory and expiratory pressure MIP and MEP, respectively). In addition tongue, strength, and endurance were assessed using the Iowa Oral Performance Instrument (IOPI) (Youmans & Stierwalt, 2006).

### 2.3 Randomization

Eligible Veterans were randomized (1:1) by a single investigator (S.M.) (using computer‐generated randomization codes) to receive oropharyngeal and RMT (intervention arm) versus sham therapy (control arm).

Sample size estimation: A formal power calculation is not appropriate as the primary purpose of the study is to evaluate the feasibility and acceptability of delivering the proposed exercise. It has been estimated that randomizing 12 participants per group will be sufficient to be able to reliably determine the primary feasibility outcomes in line with recommendations for sample sizes for feasibility/pilot trials (Julious, 2005). This number will allow us to evaluate the objectives of the trial; assess the impact of the exercise on each of the outcome measures; estimate parameters necessary to design the main trial; and enable the estimation of recruitment and retention parameters with sufficient precision.

### 2.4 Intervention

The intervention was developed for this study in collaboration with a speech and language therapist and included combined oropharyngeal exercises (involving the tongue, soft palate, and lateral pharyngeal wall) and respiratory muscle training (using resistive breathing devices). Specifically, participants in the exercise arm completed the following:

(1) Oropharyngeal training: a set of five different exercises (shown in Table 1) performed for 3 min each (15 min total duration) chosen to target the soft palate, tongue, and facial muscles as described by Guimaraes et al. and others (de Felício et al., 2018; Guimarães et al., 2009) (see oropharyngeal instruction, Supplement 1).

**TABLE 1:** Oropharyngeal and respiratory muscle exercise training steps.

	Exercise	Control
PowerBreathe	Titratable resistor	No resistor
EMST	Titratable resistor	No resistor
Tongue elevation	X	–
Tongue suction	X	–
Tongue forced downward	X	–
Tongue contraction	X	–
Soft palate exercise	X	–
Brush tongue while brushing teeth	X	–

(2) Respiratory muscle training: Two devices designed to provide resistance during the two phases of breathing were used. The “Expiratory Muscle Strength Training” (EMST) 150 device (Aspire Products LLC) was used to target expiratory muscles (Suzuki et al., 1995; Wheeler et al., 2007). The EMST was first given to the participant at its default setting (minimum resistance) and the participant was instructed to perform a quarter‐turn to increase the resistance each week and thus progressively overload the muscles (see expiratory muscle instructions, Supplement 2). The POWERbreathe Medic Plus K‐Series 3 device (POWERbreathe International Ltd., Warwickshire, UK) was used to train the inspiratory muscles. The threshold resistance for POWERbreathe was initially set at 30 cmH_2_O and the participants were instructed to increase the resistance by 5 cmH_2_O daily until they could just barely complete 30 breaths. Once they titrated up to this point, the participants were instructed to increase the resistance by 5 cmH_2_O weekly. If participants could not perform the entire 30 breaths at a given resistance, they were instructed to decrease resistance by 5 cmH_2_O (see inspiratory muscle instructions for exercise, Supplement 3). All participants were reassessed by research personnel at weeks 1 and 4 of randomization to ensure that the correct exercise technique and resistance level of the devices were being used. Each participant was asked to complete a daily log to allow for monitoring of the performed exercise duration and a weekly call was conducted with each participant to assess/confirm adherence to protocol and the well‐being of the participant. The control group performed a sham therapy which consisted of completing 30 breaths while seated using a POWERbreathe device in which the spring that provides resistance had been removed (thus providing minimal/no resistance to the respiratory muscles) (see inspiratory muscle instructions for sham, Supplement 4). All participants (in both arms) were instructed to perform the same procedure at home once a day for a 3‐month period.

### 2.5 Outcome measures

The primary outcome of this feasibility study was the success of participant recruitment, retention, adherence, acceptability, and satisfaction. The success in recruitment was assessed by measuring the percentage of participants who were interested and signed a consent document. The retention was evaluated by examining the number of participants who completed the assigned arm and 3‐month follow‐up measures. A structured follow‐up (phone interview only) was conducted by the research coordinator at 6 months (see supplement for details) to assess acceptability and satisfaction. Finally, the adherence to treatment was assessed at 3 months and considered adequate if all the exercise components were performed as follows: 30 breaths on the POWERbreathe device were performed daily, 30 breaths on the EMST, and 3 min for all tongue exercises for the exercise arm. For the sham arm, only 30 breaths on the POWERbreathe device were considered adequate adherence. Any use was defined as the number of breaths on the POWERbreathe device for the sham arm and the number of breaths on the POWERbreathe device, any number of breaths on the EMST, and any amount of time performing tongue exercises for the intervention arm. POWERbreathe adherence was verified by checking the digital records on the POWERbreathe K3 device and comparing it to the written exercise log provided by the participant. Secondary outcome measures included the Pittsburgh Sleep Quality Index (PSQI) (Buysse et al., 1991). The PSQI is a self‐rated subjective assessment questionnaire that assesses sleep quality and disturbances over a 1‐month time interval. The questionnaire has seven component scores: subjective sleep quality, sleep latency, sleep duration, habitual sleep efficiency, sleep disturbances, use of sleeping medication, and daytime dysfunction. In addition, subjects were asked to complete Fatigue Severity Scale (FSS) (Krupp et al., 1989), Insomnia Severity Index (ISI) (Bastien et al., 2001), and Epworth Sleepiness Score (ESS) (Johns, 1991). These assessment tools were in addition, PSG and physiological tests including spirometry and respiratory muscle forces were completed by participants at pre‐ and postexercise at 3‐month follow‐ups to assess the effect of the RMT exercises on subjective and objective respiratory and sleep quality.

### 2.6 Data analysis

The diagnostic study consisted of overnight polysomnography (PSG) (SomnoStar 10.2) to assess the presence and severity of SDB based on the AHI. Data from the electrooculogram, electroencephalogram, electrocardiogram, electromyogram, airflow measurement, and pulse oximeter were recorded. Respiratory events were scored and reviewed by a board‐certified sleep physician using the American Academy of Sleep Medicine (AASM) scoring manual (Berry et al., 2017). AHI was the number of events (apneas and hypopneas) per hour of sleep.

Physiological tests: The best effort of the baseline spirometry and respiratory muscle forces were reported in each participant (in both absolute and % of predicted) on the same night of the sleep study, which was then summarized as mean ± SD. Spirometry included the following variables: forced vital capacity (FVC), forced expiratory volume in the first second (FEV1), and peak expiratory flow at 25%–75% (PEF 25%–75%). The respiratory muscle forces included maximal inspiratory (MIP) and expiratory pressure (MEP). The tongue strength (Fmax, kPa) and fatigability (T‐50, seconds) were assessed using IOPI.

Sleep questionnaires were collected from participants at baseline and 3 months including ESS, FSS, ISI, and PSQI (including its components), and were summarized as mean ± SD.

### 2.7 Statistical analysis

Descriptive statistics were provided for participant demographics and sleep questionnaires (Table 2). Paired *t*–tests were used to compare participant data at baseline to follow up at 3 months, except when the normality test failed then Wilcoxon‐signed rank test was used. A value of *p* < 0.05 was considered to be statistically significant.

**TABLE 2:** Participant characteristics by group.

	Exercise	Control
Number	12	12
Age (years)	66.3 ± 10.0	64.8 ± 7.7
BMI (kg/m^2^)	29.2 ± 4.1	28.5 ± 5.6
Gender (M/F)	12/0	10/2
Race (C/A/O)	8/4/0	4/5/3
**SCI severity**		
SCI/D (C/D/T)	5/5/2	5/6/1
ASIA A	–	–
ASIA B	–	–
ASIA C	1	1
ASIA D	3	4
Multiple sclerosis	2	3
Spinal stenosis	4	3
PSQI (points)	8.8 ± 4.5	10.4 ± 5.7
ESS (points)	4.8 ± 5.0	5.9 ± 2.3
FSS (points)	29.5 ± 17.0	30.2 ± 15.1
ISI (points)	8.9 ± 6.8	13.4 ± 7.7
AHI (events/hour)	49.9 ± 22.3	44.2 ± 24.6
ODI (events/hour)	29.8 ± 22.5	15.4 ± 20.0
RAI (arousal/hour)	31.5 ± 20.7	38.5 ± 42.2
Nadir SpO_2_ (%)	82.7 ± 5.7	79.8 ± 11.1
**SDB severity**		
Mild/Moderate/Severe	0/1/11	1/4/7
Sleep efficiency (%)	77.1 ± 11.4	79.1 ± 15.2
FEV1 (%)	70.7 ± 17.9	68.1 ± 16.8
FVC (%)	74.7 ± 13.5	76.6 ± 12.3
FEV1/FVC	71.2 ± 15.8	74.3 ± 12.6
PEF 25–75 (%)	66.7 ± 24.8	62.7 ± 24.3
MIP (%)	88.5 ± 29.4	86.2 ± 30.6
MEP (%)	77.7 ± 36.2	70.5 ± 26.6
Tongue Fmax (kPa)	48.2 ± 9.3	52.6 ± 21.6
Tongue fatigability‐T‐50 (seconds)	19.9 ± 9.4	24.4 ± 16.0

*Note*: Data are presented as the mean ± S.D. There were no significant differences between the exercise and sham groups by independent *t*‐test. *p* = NS.

Abbreviations: AHI, apnea‐hypopnea index; ASIA, association of spinal cord injury classification; BMI, body mass index; ESS, Epworth Sleepiness Score; FEV1, forced expiratory volume in the first second (% predicted); FSS, Fatigue Severity Scale; FVC, forced vital capacity (% predicted); ISI, Insomnia Severity Index; MIP, maximum inspiratory pressure (% predicted); maximal expiratory pressure (% predicted); ODI, oxygen desaturation index; PEF 25–75, peak expiratory flow at 25–75%; RAI, respiratory arousal index; SpO_2_, pulse oximetry; SCI/D, spinal cord injury/disease; SDB, Sleep‐disordered breathing; SCI/D level (C) indicates cervical, (T) thoracic level of spinal cord injury, and (D) refers to the presence of spinal cord disease. Gender (M/F) refers to male and female. Race (C/A/O) refers to Caucasian, African American, and other (or unknown); PSQI, Pittsburgh Sleep Quality Index.

## 3 RESULTS

The total recruitment pool included 183 Veterans. Of these, 80 (43%) were eligible for the study and 29 (36%) agreed to participate and signed a consent.

### 3.1 Participant demographics

There were no significant differences between the exercise and sham groups at baselines when assessed using an independent t‐test (Table 2).

### 3.2 Feasibility for recruitment and retention

**FIGURE 1:**
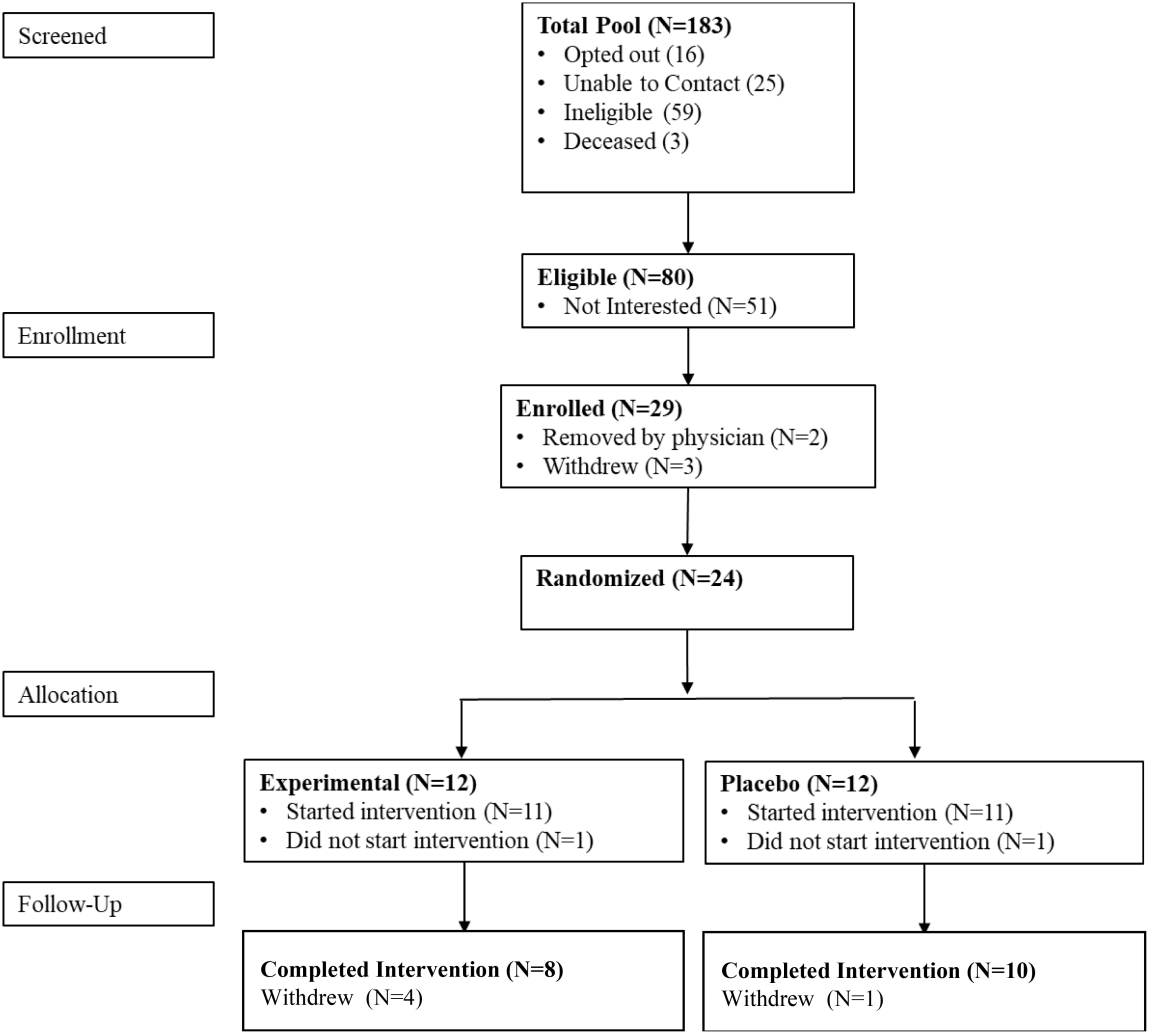
Consort chart flow depicting the recruitment and completion of the study protocol. * Two participants were removed, one due to low lumbar spinal stenosis at L4–L5 and another one due to central sleep apnea.

Twenty‐four individuals were randomized to this study protocol (Figure 1). A total of eight (67%) participants completed the 3 months of the exercise arm, and 10 (83%) participants completed the control arm. In the sham arm, one participant withdrew before starting the exercise. Four participants withdrew from the exercise arm, and one withdrew from the control arm during the exercise.

### 3.3 Adherence to the combined oropharyngeal and respiratory muscle training protocol

Table 3 summarizes the adherence data for the exercise and sham arms.

**TABLE 3:** Summary of the adherence data during 3 months of monitoring for exercise and sham arms.

Exercise			Sham		
Participant number	% days of any use	% days of adequate use	Participant number	% days of any use	% days of adequate use
1	99.6%	83.9%	1	100.0%	100.0%
2	97.6%	93.4%	2	76.3%	75.0%
3	85.5%	85.5%	3	87.2%	64.1%
4	59.2%	59.2%	4	60.0%	38.8%
5	28.3%	28.0%	5	93.1%	34.5%
6	100.0%	100.0%	6	100.0%	100.0%
7	N/A	N/A	7	96.9%	94.9%
8	N/A	N/A	8	100.0%	100.0%
			9	N/A	N/A
			10	94.9%	94.9%

*Note*: Adherence data from participants #7 and #8 in the exercise arm and #9 in the sham arms were unavailable.

Of the six participants in the exercise arm with data available to assess adherence, four (67%) reported more than 80% adherence to any use and adequate use. Two participants (33%) were not adherent (less than 80%), and two had no data available. In the control arm, seven of the ten participants (70%) who had data available reported any use, and only five (50%) had adequate use, more than 80%.

### 3.4 Acceptability and satisfaction

To assess the acceptability and satisfaction of the exercises, we reached out to all participants who completed the study for a long‐term follow‐up phone call to ask about their experiences in the study. This follow‐up phone call was done at least 6 months after enrolment in the study. Seven participants in the exercise arm were asked if they would perform the exercises if a clinician prescribed them. Six of these seven (85.7%) said that they would perform these exercises again. When asked why they would perform these exercises, their answers included “I believe it helped me,” “exercises helped to improve my voice,” and “the exercises would improve my sleep.” The one participant who said they would not perform these exercises as a treatment indicated that they would not because they “have too much on their plate.”

### 3.5 Sleep questionnaires

In comparison to baseline, there was no significant improvement in the sleep questionnaires including total PSQI and its subcomponents, FSS, ISI, and ESS at 3 months in the exercise arm, however, ISI was significantly reduced in the sham arm (as shown in Table 4).

**TABLE 4:** Secondary sleep quality questionnaire outcomes at baseline and 3‐month follow‐up for exercise and sham arms.

	Exercise			Sham		
	Baseline	3 months	*p*‐value	Baseline	3 months	*p*‐value
PSQI (points)	7.9 ± 3.3	7.0 ± 3.3	0.523	10.2 ± 5.8	8.6 ± 4.8	0.426#
Subjective sleep quality	1.1 ± 0.5	1.3 ± 0.9		1.3 ± 1.0	1.3 ± 0.8	
Sleep latency	1.3 ± 0.8	1.3 ± 1.3		1.4 ± 1.3	1.1 ± 1.2	
Sleep duration	1.1 ± 1.2	1.4 ± 1.3		1.6 ± 1.4	1.3 ± 1.3	
Habitual sleep efficiency	1.5 ± 1.2	1.6 ± 1.2		1.5 ± 1.4	1.1 ± 1.4	
Sleep disturbances	1.2 ± 0.4	1.1 ± 0.4		1.8 ± 0.8	1.3 ± 0.5	
Use of sleep medication	0.9 ± 1.3	0.3 ± 0.7		1.3 ± 1.5	1.5 ± 1.6	
Daytime dysfunction	0.9 ± 0.9	0.6 ± 0.7		1.3 ± 1.1	1.0 ± 0.7	
ESS (points)	4.8 ± 5.0	5.0 ± 4.8	0.438#	5.5 ± 2.3	4.3 ± 2.6	0.193
FSS (points)	29.5 ± 17.0	24.8 ± 12.5	0.861	31.2 ± 14.7	29.8 ± 14.9	0.645
ISI (points)*	8.9 ± 6.8	5.1 ± 5.0	0.109	12.7 ± 8.3	8.3 ± 6.2	0.040

*Note*: Data are presented as the mean ± S.D. There were no significant differences between the exercise and sham groups by Paired t‐test except ISI in sham. (* There are two missing measurements at baseline in the exercise and three missing in the sham). Paired t‐test was used except when the normality test failed then the Wilcoxon‐signed rank test was used (#).

Abbreviations: ESS, Epworth Sleepiness Score; FSS: Fatigue Severity Scale; ISI, Insomnia Severity Index; PSQI, Pittsburgh Sleep Quality Index.

### 3.6 Physiological and sleep tests

**FIGURE 2:**
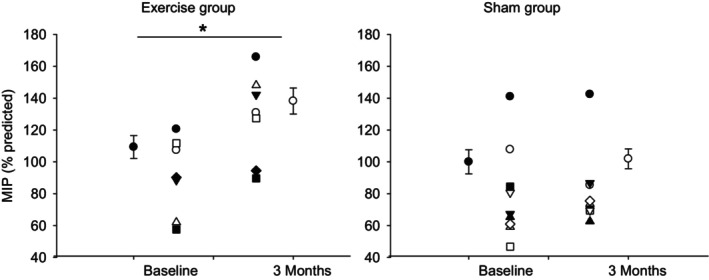
Maximal inspiratory pressure (MIP) at baseline and after 3 months of exercise or sham. * *p* < 0.05 vs. baseline using paired Student's *t*‐test. Data are presented as individual values and mean ± SE.

**FIGURE 3:**
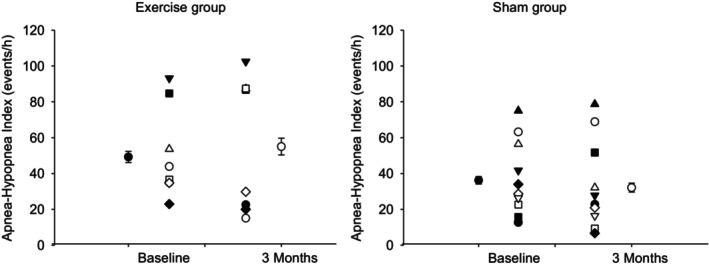
Apnea‐hypopnea index (AHI) at baseline and after 3 months of exercise or sham. Data are presented as individual values and mean ± SE.

There was no significant difference using at baseline between the two groups in spirometry function (FEV1 and FVC), respiratory muscle forces (MEP and MIP), or tongue pressure (see Table 2). No significant change was noted at the end of 1‐ and 3‐month periods in MEP, FEV1, FVC, or tongue pressures in either the exercise or control groups (Table 5). However, it is important to point out that 4/8 participants in the exercise arm had FEV1/FVC < 0.7 indicating the presence of airflow obstruction, and 1/10 had FEV1/FVC < 0.7 in the sham arm. FEF25‐75 was reduced in the exercise group (60% of predicted) and did not change significantly (79% of predicted; *p* = 0.112) 3 months following RMT. Likewise, PEF25‐75 did not change in the sham arm at 3 months compared with baseline (59% vs. 68%; respectively, *p* = 0.346). At the end of the 3‐month period, MIP was significantly increased (*p* < 0.05) in the exercise group compared with baseline, and no significant change was noted in the sham group (as illustrated in Figure 2). No significant change was noted at the end of 3‐month periods in AHI or oxygen desaturation index in either the exercise or sham groups (Figure 3).

**TABLE 5:** Secondary physiological outcomes at baseline and 3‐month follow‐up for exercise and sham arms.

	Exercise			Sham		
	Baseline	3 months	*p*‐value	Baseline	3 months	*p*‐value
FEV1 (%)	67.8 ± 20.9	78.3 ± 17.4	0.239	68.1 ± 16.8	69.1 ± 22.4	0.652#
FVC (%)	78.0 ± 11.4	79.3 ± 7.7	0.597	76.6 ± 12.3	73.4 ± 20.6	0.313#
FEV1/FVC	64.1 ± 12.2	70.7 ± 14.6	0.844#	76.2 ± 13.1	70.9 ± 12.2	0.382
PEF 25–75 (%)	59.7 ± 27.6	79.3 ± 13.5	0.112	62.7 ± 24.3	58.9 ± 26.9	0.346
MIP (%)	91.1 ± 24.3	128.3 ± 27.8*	0.011	83.2 ± 30.6	81.7 ± 24.1	0.661
MEP (%)	87.7 ± 34.2	97.3 ± 36.6	0.419	70.5 ± 26.6	66.8 ± 27.8	0.575
*F*_max_ (kPa)	49.9 ± 4.1	48.0 ± 8.0	0.426	48.2 ± 14.2	49.2 ± 10.9	0.742
T‐50 (s)	16.1 ± 8.3	24.0 ± 13.8	0.177	24.3 ± 10.6	32.4 ± 36.1	0.652#

*Note*: Data are presented as the mean ± S.D. There were no significant differences between the baseline and 3 months in either group except MIP increased significantly in the exercise group (*). Paired *t*‐test was used except when the normality test failed then the Wilcoxon‐signed rank test was used (#).

Abbreviations: FEV1, forced expiratory volume in the first second (% predicted); *F*_max_: Tongue maximal pressure (kPa) at bedtime; FVC, forced vital capacity (% predicted); MIP, maximum inspiratory pressure (% predicted); maximal expiratory pressure (% predicted); PEF 25–75, peak expiratory flow at 25%–75%; T‐50, tongue endurance at bedtime (seconds).

## 4 DISCUSSION

This study demonstrated the feasibility and acceptability of combined home‐based oropharyngeal and respiratory muscle training (RMT), evidenced by high recruitment, adherence, and retention. These favorable feasibility findings for the overall training program were supported by highly positive responses 6 months later. In addition, the 3‐month exercise arm was associated with significant improvement in maximal inspiratory muscle forces. In contrast, the effects on other physiological parameters, sleep apnea severity, and symptoms of excessive daytime sleepiness and fatigue were not seen postexercise or at follow‐up at 3 months. This may be explained by the low levels of hypersomnia and fatigue symptoms observed at baseline among our participants, severity, or heterogeneity of the disease at baseline.

The literature regarding the feasibility of respiratory muscle training on respiratory function in chronic respiratory disorders such as COPD and esophageal cancer (Hulzebos et al., 2006; Sturdy et al., 2003). For example, Sturdy et al. conducted a high‐intensity interval‐based RMT for patients with severe COPD over an 8‐week period (including three 20‐min sessions per week). The study concluded that high‐intensity training that targeted inspiratory muscles was feasible and resulted in a 32 ± 27% increase in MIP (Sturdy et al., 2003). However, Sturdy et al.'s study performed all the RMT under supervision and was not home‐based which limits its generalizability for individuals with SCI who have mobility limitations.

Previous investigators also assessed the feasibility of RMT in individuals with SCI (Kang et al., 2022; McDonald & Stiller, 2019). For example, McDonald et al. found that a high‐resistance, with a low‐repetition program of inspiratory muscle training using a POWERbreathe KH1 device, was safe and feasible in individuals with acute cervical and thoracic SCI (McDonald & Stiller, 2019). However, this study was not randomized nor it was controlled for a short duration and the number of sessions was 50 sessions over 10 days only. Likewise, Kang et al. used game‐based RMT twice weekly, for 8 weeks in individuals with chronic cervical SCI and found the training exercises were feasible and improved pulmonary function and muscle forces MIP and MEP (Kang et al., 2022). However, Kang et al. study did not assess for sleep apnea in their participants and was not a controlled study.

The literature regarding the efficacy of respiratory muscle training on sleep symptoms/quality remains inconclusive. For example, Kuo et al. conducted a respiratory exercise trial for 5 weeks using EMST (Kuo et al., 2017). They demonstrated improved PSQI scores in the EMST arm compared to the control arm; however, these mostly occurred in the moderate to severe OSA group and were not notable in the mild OSA group. Another study found that RMT decreased the rate of respiratory complications within 3–12 months of treatment initiation in patients who had an ischemic stroke (Oliveira et al., 2018). There is also evidence that combined inspiratory muscle and oropharyngeal training can improve fatigue, sleepiness, and quality of life in patients with stable but untreated OSA (Erturk et al., 2020). Previous studies on participants with SCI have demonstrated that the majority have daytime sleep symptoms and fatigue, while RMT use may result in improvements in sleep quality (Russian et al., 2011). These findings did not agree with our results showing that sleep quality did not improve in the exercise arm after 3 months of combined oropharyngeal and respiratory muscle exercises or in the control arm. A possible explanation for this may be that the combined exercise strategies improved respiratory muscle strength and lung volumes during wakefulness but not during sleep especially since the severity of OSA did not change in either group.

Previous studies in able‐bodied participants showed that oropharyngeal exercise improves the severity of moderate OSA and symptoms such as snoring and sleepiness (Guimarães et al., 2009). Our study did not show improvement in the severity of OSA, which might be explained by the fact the population is different including SCI/D and were more severe in the exercise and sham group (AHI 52.9 ± 26.6 and 37.0 ± 21.7 events/h, respectively) while the baseline AHI for the previous studies was moderate (22.4 ± 4.8 events/h).

### 4.1 Clinical implications

The combined training program is simple, feasible, and acceptable to individuals with SCI/D. While a significant increase in MIP was achieved in the exercise arm, other physiological and sleep indices did not significantly improve. The long‐term effect of the combined exercise program in SCI/D and therapeutic use for OSA remains to be determined. Previous studies using RMT showed improved pulmonary function indices, particularly in those with obstructive lung disease (Belman et al., 1980; Degre et al., 1974; Sonne & Davis, 1982) (COPD). In this study, there was a trend for improved peak expiratory follow (an indicator for small airway obstruction) in the exercise arm while no change in the control suggesting a potential role in this group of patients who may have obstructive airway disease also.

### 4.2 Methodological considerations

Our study has a few methodological considerations to point out. First, patients had moderate‐to‐severe OSA (AHI ≥ 15), with both SCI and disease, hence, there was heterogeneity of spinal cord involvement that could affect the response to RMT. The sample was predominantly men which is likely a result of our participant population being Veterans. Second, although most participants (~ two‐thirds) reported a high level of adherence and use of the combined exercise, the exercise was performed at home without direct observation and the level of resistance was not recorded from each participant at each measurement time point as the resistance changed manually (see supplements for instruction). However, each participant was provided individual one‐on‐one instruction with follow‐up education within the first months. Third, we did not restrict recruitment to one severity of OSA or PAP treatment. This may have affected the results of the study including sleep quality questionnaires and PSG. However, only four participants assigned to the exercise and 2 participants assigned to the control arm were on PAP before the treatment and they were asked to keep the same level of use throughout the study. PAP adherence (>4 h average daily use for the duration of the study) was 35% and 33% in exercise and control arms, respectively, suggesting similar PAP adherence between groups. Finally, the sample of participants was small and heterogeneous including SCI/D at different levels and severity. Nevertheless, most participants demonstrated tolerance and acceptance of the exercise.

## 5 CONCLUSIONS

Implementing a home‐based combined oropharyngeal and respiratory muscle training program was feasible and acceptable, with a reasonable level of adherence among individuals with OSA and SCI/D. Therefore, further investigations should be performed to determine its efficacy in improving respiratory and sleep quality in individuals with SCI/D, who struggle to adhere to other treatment options.

## AUTHOR CONTRIBUTIONS

All authors have seen and approved the manuscript. All authors have agreed to be accountable for all aspects of the work.

## FUNDING INFORMATION

Dr. Sankari was supported by (U.S.) Department of Veterans Affairs (V.A.) Award from RR&D (RX002885). Dr. Badr was supported by the Department of Defence #SC150201, Department of Veterans Affairs, Merit Review #1I01CX001040, and National Heart, Lung, Blood Institute #R01HL130552.

## CONFLICT OF INTEREST STATEMENT

The authors have no potential conflict of interest to disclose.

## ETHICS APPROVAL STATEMENT

The Human Investigation Committees of the Wayne State University School of Medicine and the John D. Dingell Veterans Affairs Medical Canter approved the experimental protocols.

## THE INSTITUTION WHERE WORK WAS PERFORMED

John D. Dingell VA Medical Center from November 2018 to October 2021.

## Supporting information


Data S1:
