# Supplementary material for: Feasibility of oropharyngeal and respiratory muscle training in individuals with OSA and spinal cord injury or disease: A pilot study
Source: Physiol Rep. 2024 Feb 7;12(3):e15930. doi: 10.14814/phy2.15930 (PMC10849885; doi:10.14814/phy2.15930)
Supplement: Supplementary file 1 — Data S1: [file PHY2-12-e15930-s001.docx]

**Supplement**

**OROPHARYNGEAL TRAINING: Subject Instructions**

TONGUE BRUSHING:

Brush tongue when brushing teeth.

TONGUE MOVEMENT:

1. TONGUE ELEVATION: a. Raise the tip of your tongue to the top of your mouth and keep the rest of it down. Perform a wave-like motion that ends with the tip of the tongue behind the bottom front teeth and the back of your tongue is in contact with the top of your mouth. Part of your tongue should be in contact with the roof of your mouth at all times. b. Perform this motion continuously for 3 minutes a day.

2. FORCED TONGUE SUCTION INPALATE: a. Elevate your tongue to the roof of your mouth. Retract it backwards to create a suction for as long as you can. When your tongue slips and the suction is lost, perform this action again. b. Complete for 3 minutes a day, focusing on keeping suction as long as you can.

3. FORCED BACK OF TONGUE DOWNWARD: a. Elevate the front of your tongue against the roof of your mouth and concentrate on pushing the back of your tongue down for as long as you can. If your tongue falls out of place, repeat the motion. b. Complete for 3 minutes a day.

4. TONGUE CONTRACTION: a. Place tip of tongue behind lower teeth. b. Create a valley in the center of the tongue and lift both sides of the tongue and hold this position for as long as you can. c. Complete for 3 minutes a day.

SOFT PALATE:

1. Say “ah” a few times and note the soft palate tensing and relaxing near the back of the roof of your mouth. Allow soft palate to relax between each “ah”.

2. Say “ah” and HOLD soft palate and muscles tight, midsound. Keep these muscles tight as long as you can. When they become loose, perform this motion again.

3. Complete step 2 for 3 minutes a day

**Expiratory Muscle Strength Trainer  EMST 150 - Participant Instructions**

Schedule:

- Complete 5 days a week, 5 sets with 5 repetitions per set (25 total breaths) each day.
- Training will take about 20-30 minutes/day.
- Try to do exercises at the same time each day.

How the device works:

- This device helps to strengthen muscles you use to breathe air out
- When you breathe out with enough pressure a spring loaded valve will open and air will move easily.  If you do not breathe out with enough pressure the valve will not open.
- To adjust valve difficulty:
  - Turn valve clockwise to make it more difficult to breathe out.
  - Turn the valve counterclockwise to make it easier to breathe out.
- Your first valve setting will be determined during the first session.
- Do NOT adjust the valve until after you have trained for 1 week.

First Week of Training:

1. Make sure device is set to _______________
   1. To adjust device: turn knob until the metal screw lines up with ____________
2. Place nose clips on nose
3. Take a deep breath in and hold it
4. Place mouthpiece in your mouth, behind your teeth.
5. Secure your lips tightly around mouthpiece.
   1. Hold/press the sides of your cheeks if needed.
6. Breathe out hard and fast using your chest and stomach muscles to push air through the device. This breathing effort should only last a couple seconds for the air to move through the device.
7. Rest for a minimum of 15-30 seconds between each breath.
8. Do NOT skip resting in between breaths. Your muscles need time to prepare for the next breath.
9. Complete exercise 5 times with 15-30 second rest between each breath.  After every 5th breath rest for 1 minute (this is 1 set).
10. Repeat 4 more times for a total of 5 sets, 25 total repetitions.
11. STOP if you feel lightheaded at any time during the exercise.
12. Record date/time of exercise completion.

Week 2-5 Training:

1. Turn knob 1/4 turn clockwise at the beginning of week 2.
2. Increase resistance, (gradually turn the knob clockwise) as long as you can still move air easily through the device without way too much effort.
3. Complete exercises 3 days a week, 5 sets of 5 repetitions and same breaks, 15-30 seconds between repetitions and 1 minute break between each set.

- Write down the date/time and resistance level.

**Powerbreathe MedicPlus - Participant Instructions Control (Sham Group)**

Schedule:

- Use device twice a day
- 30 breaths each time
- Pause 3-4 seconds between breaths, until you feel the need to breathe in again.

To use the device:

1. Place PowerBreathe device mouthpiece in your mouth so that your lips cover the outer shield to make a seal.  Mouthpiece bite blocks are gripped between your upper and lower teeth.
2. Place nose-clip on nose.
3. Breathe out as far as you can and then take a FAST hard breath in through your mouth.  Take in as much air as you can, as quickly as you can, straightening your back and expanding your chest.
4. Breathe out slowly and easily through your mouth until your lungs are empty.  Let the muscles in your chest and shoulders relax. Pause 3-4 seconds until you feel the need to breathe in again.
5. Repeat until you feel good about breathing through the device. Continue to practice taking a strong breath in and then breathing out slowly and fully.

Training Schedule (Weeks 1-6):

1. Complete 30 breaths twice a day
2. Keep a journal to write down when and how much you use the device.

Maintenance training after week 6:

- Use device 30 breaths in a row, twice a day, every day.
- Keep a journal to write down when and how much you use the device.

You may notice that it is harder to completely fill your lungs during training sessions.  This is because your breathing muscles are getting tired.  If you feel out of breath, lightheaded or feel the need to cough, take a short break.  When you feel better continue session until you have completed 30 breaths.

**Powerbreathe MedicPlus - Participant Instructions**

Schedule:

- Use device twice a day
- 30 breaths each time
- Pause 3-4 seconds between breaths, until you feel the need to breathe in again.

How the device works:

- This device is designed to exercise the muscles you use to breathe in.
- It should be hard to breathe in but not to breathe out.

To use the device:

1. Place PowerBreathe device mouthpiece in your mouth so that your lips cover the outer shield to make a seal.  Mouthpiece bite blocks are gripped between your upper and lower teeth.
2. Place nose-clip on nose.
3. Breathe out as far as you can and then take a FAST hard breath in through your mouth.  Take in as much air as you can, as quickly as you can, straightening your back and expanding your chest.
4. Breathe out slowly and easily through your mouth until your lungs are empty.  Let the muscles in your chest and shoulders relax. Pause 3-4 seconds until you feel the need to breathe in again.
5. Repeat until you feel good about breathing through the device. Continue to practice taking a strong breath in and then breathing out slowly and fully.

Training for weeks 1-6:

1. Complete 30 breaths twice a day
2. Select a resistance level that is as hard as possible without causing discomfort
3. Day 1: set load to 0 and try to complete a 30 breath cycle. Turn knob clockwise to increase resistance (make it harder).  Turn knob counter clockwise to decrease resistance (make it easier).
   1. If you are unable to complete 30 breaths in a row, take a short rest and start again until you have completed 30 total breaths. Remain at load 0 for 1 week (30 breaths, twice a day)
   2. If it was easy to complete 30 breaths, increase load level by 1 each day, until you are only just able to complete 30 breaths.
   3. Once you have reached this point, complete 30 breaths in a row, twice a day for 1 week.
4. Increased load by 1/2 turn a week.
5. Keep a journal to write down your progress including date and load level.

Training after week 6:

- Use device 30 breaths in a row, twice a day, every day at the same resistance level.

**Six Month follow up phone call and follow up questions**

Would you be interested in participating in a similar sleep research study involving exercises similar to the ones that you performed?

Why/why not?

If exercises similar to the ones you were performing were given to you as a treatment for sleep apnea, would you be willing to perform them?

Why/why not?
